# A Case of Severe Neuroleptic Malignant Syndrome in a Patient with Chronic Schizophrenia

**DOI:** 10.1192/j.eurpsy.2025.2080

**Published:** 2025-08-26

**Authors:** P. Ibáñez Mendoza, Á. de Vicente Blanco, B. Orgaz Álvarez, M. Velasco Santos, C. Herranz Serfaty, V. Giunti, L. Nocete Navarro

**Affiliations:** 1 Psychiatry, Hospital Universitario La Paz, Madrid, Spain

## Abstract

**Introduction:**

Neuroleptic Malignant Syndrome (NMS) is a rare, idiosyncratic, and potentially lethal reaction to psychopharmacological treatment. Several heat-related disorders are influenced by psychopharmacological use, making differential diagnosis challenging. As there are no specific clinical or laboratory findings for NMS, excluding other causes of hyperthermia is essential for accurate diagnosis.

**Objectives:**

To describe a case of severe NMS and review the key elements of differential diagnosis and treatment options.

**Methods:**

A case report and a non-systematic review of the literature.

**Results:**

A 54-year-old male was admitted to the hospital after being found unconscious on the street during a hot August day, with apparent cranioencephalic trauma and a core temperature of 41°C. The patient had a known history of chronic schizophrenia, managed with risperidone (6 mg/day) and quetiapine (800 mg/day), with no recent medication changes. Initial examination in the Emergency Department revealed tachycardia, tachypnea, normotension, diaphoresis and confusion. Laboratory results showed mild leukocytosis with neutrophilia, hyperkalemia, hypernatremia, and elevated acute phase reactants, including a creatine kinase (CK) level exceeding 3000 IU/L. Lactic acidosis and impaired renal function (creatinine 1.9 mg/dL) were also noted. Infectious causes were ruled out, and neuroimaging did not reveal any acute findings to account for the symptoms. Due to persistent decreased consciousness, the patient required intubation and supportive care in the Intensive Care Unit (ICU).

The patient exhibited persistent hyperthermia that was unresponsive to antipyretics and physical cooling measures. Upon suspicion of NMS, antipsychotic medications were discontinued, and treatment with a dopamine agonist and dantrolene was initiated. Muscle rigidity, which developed later, further supported the NMS diagnosis. Electroencephalography (EEG) findings were consistent with NMS. Given the patient’s slow recovery, electroconvulsive therapy (ECT) was started but discontinued after two sessions due to ICU-related infectious complications.

After 19 days of hospitalization and treatment, the patient showed significant clinical improvement, allowing for extubation and discontinuation of intensive care interventions.

**Conclusions:**

This case emphasizes the importance of early recognition and treatment of Neuroleptic Malignant Syndrome to avoid complications and mortality. Differentiating NMS from classical heat stroke can be challenging, with muscle rigidity serving as a critical diagnostic feature.

**Disclosure of Interest:**

None Declared

